# ELAVL Proteins, miRNA Fate, and Extracellular RNA Communication in Brain Aging and Neurodegeneration

**DOI:** 10.3390/cells15181649

**Published:** 2026-09-11

**Authors:** Kamalika Mukherjee, Suvendra N. Bhattacharyya

**Affiliations:** 1 Department of Anesthesiology, University of Nebraska Medical Center, Omaha, NE 68198, USA; 2 Department of Pharmacology and Experimental Neuroscience, University of Nebraska Medical Center, Omaha, NE 68198, USA

**Keywords:** ELAVL, HuD, HuR, miRNA fate, extracellular vesicles, brain aging, neurodegeneration, neuron–glia communication

## Abstract

**Highlights:**

**What are the main findings?**

- ELAVL proteins connect intracellular miRNA regulation with extracellular RNA communication.
- HuD promotes let-7a and miR-125b export during neuronal differentiation.
- HuR regulates selected miRNAs through intracellular buffering and endosomal export.
- ELAVL–miRNA–EV signaling in brain aging and neurodegeneration remains a testable framework.

**What are the implications of the main findings?**

- ELAVL proteins may determine whether selected miRNAs remain active, are buffered, or are exported in EVs.
- Altered HuD/HuR activity could disrupt neuronal RNA homeostasis and neuron–glia communication.
- This framework may explain the context-dependent protective and pathogenic effects of HuD.
- Selective ELAVL–miRNA interactions may offer future therapeutic targets in neurodegeneration.

**Abstract:**

Brain aging is accompanied by changes in RNA homeostasis, intercellular communication, and stress responses that increase vulnerability to neurodegenerative diseases. The neuronal protein HuD (ELAVL4) and the broadly expressed HuR (ELAVL1) regulate RNA stability, translation, localization, and selected microRNA (miRNA) activities. Recent studies further show that HuD can promote export of let-7a and miR-125b during differentiation of PC12 cells, whereas HuR can bind selected miRNAs and engage endosomal export or intracellular buffering mechanisms in other cellular contexts. These observations support an emerging ELAVL–miRNA–extracellular vesicle (EV) framework. They do not yet establish an integrated HuD/HuR-dependent neuron–glia pathway in aging human brain or Alzheimer’s disease (AD). We therefore distinguish direct, model-specific findings from hypotheses that age-, amyloid-, or inflammation-associated changes in ELAVL abundance, localization, or RNA binding could redistribute miRNAs between intracellular pools and EVs. Testing this framework in primary human neural cells, induced pluripotent stem cell-derived systems, organoids, and *in vivo* models may identify context-specific mechanisms and therapeutic opportunities.

## 1. Introduction

Neuronal ELAVL4, also known as HuD, is a crucial RNA-binding protein indispensable for neuronal development, plasticity, and survival [1]. Its dysregulation, whether through gain or loss of function, has been associated with neurodegenerative disorders such as Alzheimer’s disease (AD), Parkinson’s disease (PD), and amyotrophic lateral sclerosis (ALS) [2]. HuD influences RNA metabolism at multiple stages: it stabilizes messenger RNA (mRNA) and promotes translation by binding to AU-rich elements in targets including GAP-43 [3], BDNF [4], and tau [5]; functions as a splicing factor to generate neuron-specific isoforms; modulates polyadenylation to produce longer 3′ untranslated regions (3′ UTRs) [6]; transports mRNAs to axons and growth cones [7,8]; and represses the translation of targets such as p27 and Ins2 [9]. Recent research indicates that HuD has a dual role in neurons. HuD depletion causes defects in neuronal differentiation and synaptic physiology [10], whereas elevated levels may activate pathogenic pathways [11,12,13]. Recently, researchers found that under stress, HuD is sequestered by Y RNAs, impairing its mRNA binding [14]. In NGF-differentiated PC12 cells, HuD associates with let-7a and miR-125b and promotes their extracellular vesicle export, coincident with reduced intracellular activity of these miRNAs; whether this mechanism operates in primary neurons or contributes to neuronal survival in vivo remains to be established [15]. These findings suggest that targeted modification of HuD’s RNA-binding capabilities may reduce aberrant gene expression and regulation associated with AD, PD, HIV-associated neurocognitive disorders (HAND), or the aging process in the brain (Table 1). Here, we discuss key mechanisms of HuD-mediated RNA regulation reported in neurons and their possible crosstalk with other nELAVLs and HuR in the aging or degenerating brain.

## 2. HuD and HuR as Regulators of Post-Transcriptional Events

**Figure 1:**
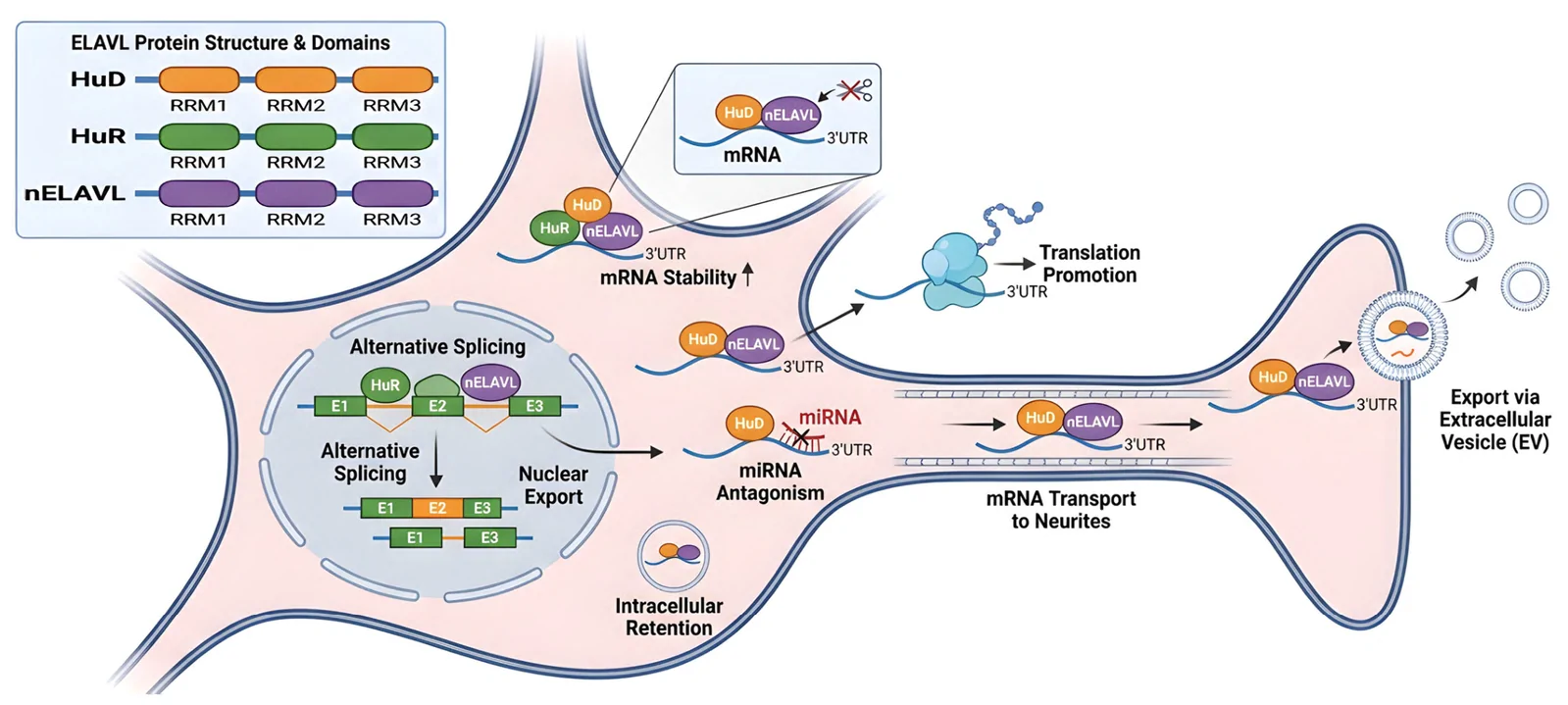
**ELAVL proteins as regulators of neuronal RNA fate.** The figure depicts ELAVL proteins—HuD, HuR, and other neuronal ELAV-like proteins (nELAVLs; i.e HuB or HuC)—as pivotal regulators of neuronal RNA fate. It summarizes their domains and cellular distribution, emphasizing their roles in mRNA stability, translation, splicing, and localization. It also illustrates ELAVL–miRNA interactions, intracellular miRNA retention, and miRNA export via extracellular vesicles. Together, these processes show how ELAVL proteins coordinate intracellular and extracellular RNA fate. Arrows indicate the direction of RNA processing, transport, or export; HuD, HuR, and other nELAVLs are color-coded as shown in the inset.

**Elav in Drosophila is a key RNA-binding protein in neurons, crucial for nervous system development.** It regulates alternative splicing and polyadenylation, essential for neural-specific mRNAs [19,20]. In mammals, the ELAVL protein family, especially the ubiquitously expressed ELAVL1 (HuR), regulates mRNA stability and translation in diverse cell types. HuR is involved in stress responses, controlling mRNA stabilization and miRNA activity [21,22]. Recent research shows that HuR mediates miRNA regulation, including their export via extracellular vesicles (EVs) and storage in organelles such as mitochondria [23,24,25]. HuR can reversibly bind to miRNAs, facilitating their export via EVs or organellar storage [23,26]. Neuronal ELAVL (nELAVL) proteins HuB, HuC, and HuD are primarily expressed in neurons but are less well studied as regulators of gene expression [1]. Some studies support roles for HuC and HuB in brain RNA regulation, while HuD is known for mRNA stabilization, splicing, transport, and miRNA regulation in brain cells [6] (Figure 1).

### 2.1. Expression Regulation of HuD in Neurons

Understanding how molecular processes control neuron-specific HuD expression is key to clarifying HuD-mediated RNA functions. Studies show that regulation occurs at the transcriptional, post-transcriptional, and post-translational levels, ensuring that HuD is expressed specifically in neurons. Transcriptional regulation involves factors such as Ngn2, which binds to E-boxes in the HuD promoter to promote HuD transcription [27], and SATB1, a HuD mRNA target that also enhances HuD transcription in a positive feedback loop, thereby stabilizing and increasing HuD transcript levels [28]. Signaling pathways such as Notch3 and insulin, via the IR–IRS–Akt–FoxO1 axis, also increase HuD expression [9,29], whereas T3 hormone inhibits HuD transcription [30].

Alternative splicing generates diverse HuD isoforms [31], whereas miRNAs such as miR-375 and miR-129-5p suppress HuD by destabilizing its mRNA and blocking translation [32]. Celf1 binds HuD’s 5′ UTR, hindering translation [33]. Post-translational modifications, such as CARM1 methylation, decrease HuD stability [34], whereas PKCα phosphorylation stabilizes HuD mRNA [35], thereby influencing neuronal differentiation and gene regulation [36]. HuR reciprocally regulates HuD, primarily repressing it post-transcriptionally [15]. Although several regulatory factors are known, further research is needed to elucidate the mechanisms underlying HuD regulation [1,15].

### 2.2. Genes and Pathways HuD Regulates in Neurons

Since HuD was first identified in the brain, it has been recognized as a key regulator of post-transcriptional neuronal gene expression, documented in both invertebrates and vertebrates (reviewed in [37]). It stabilizes neuronal mRNAs, such as growth-associated protein 43 (GAP-43) mRNA, thereby supporting neurite growth [38,39]. HuD regulates the post-transcriptional control of key neuronal mRNAs, including BDNF [40], NGF, NT–3 [41], Nova1 [42], neuritin 1 [43], neuroserpin [44], AChE [45], and SATB1 [28]. These functions impact neuronal differentiation, neurogenesis, dendritic development, plasticity, synaptic communication, and signaling pathways. HuD modulates mRNAs involved in neurodegenerative diseases and cancer, including APP, BACE1, BACE1AS lncRNA [46], neprilysin (NEP) [47], tau [5,48], SOD1 [5], and MYCN [49,50]. HuD stabilizes mRNAs such as p21 [51], CaMKIIα [52], and MSI1 [53] in non-brain and brain tissues. The circular RNA CirHomer1a, derived from neuronal development- and synaptic plasticity-associated genes, is also a HuD target [54]. HuD enhances gene expression by increasing the translation of targets such as Nova1 [55], Kv1.1 [56], and mTORC1-regulated genes in neurons [55]. In addition to influencing mRNA stability and translation, HuD also participates in the alternative splicing and polyadenylation of pre-mRNAs, including those encoding CGRP, NF1, APP, and glutaminase, as demonstrated in the mouse neocortex [6,46,57,58].

### 2.3. Mechanism of HuD-Mediated Gene Regulation in Neurons

HuD regulates neuronal gene expression primarily via post-transcriptional mechanisms. Acting as a neural translation enhancer, HuD influences mTORC1 pathways through its three RNA-recognition motifs (RRMs); RRMs 1 and 2 target AREs in 3′ UTRs, and RRM 3 binds the poly(A) tail [55,59]. HuD increases mRNA stability, extending the half-life of transcripts such as GAP-43, BDNF, and CaMKIIα, by competing with decay factors AUF1 and TTP [35,52,60]. It promotes translation by interacting with the initiation factor eIF4A and PABP, thereby aiding ribosome attachment [61]. In some contexts, it represses translation, for example by binding to the p27 mRNA IRES [62,63]. HuD also influences alternative splicing and polyadenylation, creating diverse isoforms or stable transcripts [6,46,57]. It directs mRNAs to specific cellular regions, such as axons and dendrites, sequestering them within granules during neuronal activity [10,35]. Synaptic activity reorganizes HuD–RNA interactions, stabilizes mRNAs, and shifts them from storage to translation—supporting localized protein synthesis, which is vital for synaptic tagging and long-term memory [40,64].

## 3. Neurodegenerative Disease Relevance of HuD Alteration in Neurons

Multiple studies link HuD to neurodegenerative disorders such as AD, PD, and ALS (reviewed in [2]). AD is associated with memory loss and cognitive decline, while amyloid deposition and plaque formation contribute to neuroinflammation. There is currently no cure for Alzheimer’s disease; however, treatments are available to help manage symptoms, and some newer medications may slow disease progression [65]. Elevated HuD levels in AD brains may contribute to the disease by increasing mRNAs involved in amyloid–β formation, such as APP and BACE1 [66]. Another study found reduced nELAV levels in the AD hippocampus and decreased HuD levels after Aβ42 treatment in SH-SY5Y cells [66]. Mouse models with HuD deletion demonstrate HuD’s role in AD, as HuD deletion improves cognition in these models [12]. These divergent findings on HuD levels in AD likely stem from differences across the brain regions studied, underscoring the need for further research to determine HuD’s precise role in AD.

Parkinson’s disease (PD) is a progressive neurodegenerative disorder that results from the loss of dopamine-producing neurons, leading to reduced dopamine availability. No cure exists, but treatments such as levodopa and deep brain stimulation (DBS) help manage symptoms. In PD, multiple SNPs in the HuD gene, such as rs967582, rs2494876, and rs3902720, are associated with symptom onset, but their effects on HuD protein levels or on target mRNA affinity remain unclear [67].

ALS, or Lou Gehrig’s disease, is a progressive neurodegenerative disease that destroys motor neurons controlling voluntary muscles. There is also no cure, but treatments can help manage symptoms. Recent studies show increased HuD levels in human iPSCs carrying the FUS P525L mutation associated with ALS [13], and higher HuD levels in the ALS motor cortex correlate with increased SOD1 mRNA [68].

## 4. The Impact of Amyloid Proteins on miRNA Pathways and the Possible Involvement of ELAVLs in Regulating Neuroinflammation

Amyloid-β (Aβ) accumulation in Alzheimer’s disease causes neural damage by disrupting microRNA (miRNA) regulation and triggering neuroinflammation [69,70]. Amyloid proteins hinder miRNA function by sequestering miRNAs in P-bodies, thereby interfering with miRNA-mediated gene repression [18,70]. This also affects RNA-binding proteins such as Ago and ELAVLs, which are important in inflammatory and amyloid-related pathways. Aβ peptides alter miRNA levels and processing, thereby impacting gene regulation linked to AD [71,72]. Amyloid reduces protective miRNAs such as miR-101, miR-106b, and miR-31, and increases disease-associated ones, promoting further amyloidogenesis [73]. This hampers BACE1/β-secretase regulation, as miRNAs such as miR-29a/b1, which are expected to inhibit BACE1, are reduced in AD [74]. Aβ toxicity also reduces Argonaute-2 (Ago2), a critical miRISC component, thereby impairing miRNA function [75]. miR-31 improves cognition and abolishes amyloid-β pathology by targeting APP and BACE1 in an animal model of AD [76]. Additionally, Aβ disrupts the processing of primary miR-650, thereby affecting the APOE, PSEN1, and CDK5 pathways [77]. The increase in non-functional miRNPs, such as miR-146a miRNPs, in the AD brain arises from their storage in RNA processing bodies, which can be recycled via activation of the Rheb–mTORC1 pathway and P-body recycling, as demonstrated in the rodent brain with cognitive recovery [70].

ELAVL proteins, including neuronal HuD and broadly expressed HuR, are vital for mRNA stability and translation during neuroinflammation. In the prefrontal cortex of AD models, HuR levels increase, and HuR binds to APP, promoting cell death [78]. HuR is activated in reactive microglia, stabilizing cytokine mRNAs such as IL-1 and TNF-α, and silencing HuR reduces inflammation [79]. HuR could also export miRNAs from amyloid-exposed astroglia, possibly via EVs, to neurons, thereby exacerbating inflammation [80]. Conversely, EV export of anti-inflammatory miRNAs from glia might limit neuroinflammation, though the mechanism by which HuR balances these processes remains unclear.

Neuronal ELAVLs, especially HuD, decline in the AD hippocampus, which is linked to higher Aβ levels [66]. HuD stabilizes ADAM10 mRNA encoding a non-amyloidogenic enzyme, but Aβ disrupts this stabilization, thereby promoting amyloid buildup [81]. Reduced HuD activity leads to abnormal splicing of AD genes, resulting in toxic variants [82]. One hypothesis suggests that Aβ worsens neuroinflammation by shifting HuR toward a pro-inflammatory role in glia and reducing neuronal HuD, thereby affecting neuronal health. HuD’s roles in miRNA export and neuroinflammation, and HuR’s influence on cytokine mRNAs in AD, are unclear.

## 5. Intercellular Communication Between Neurons and Glial Cells via miRNAs and the Role of ELAVL Proteins

**Figure 2:**
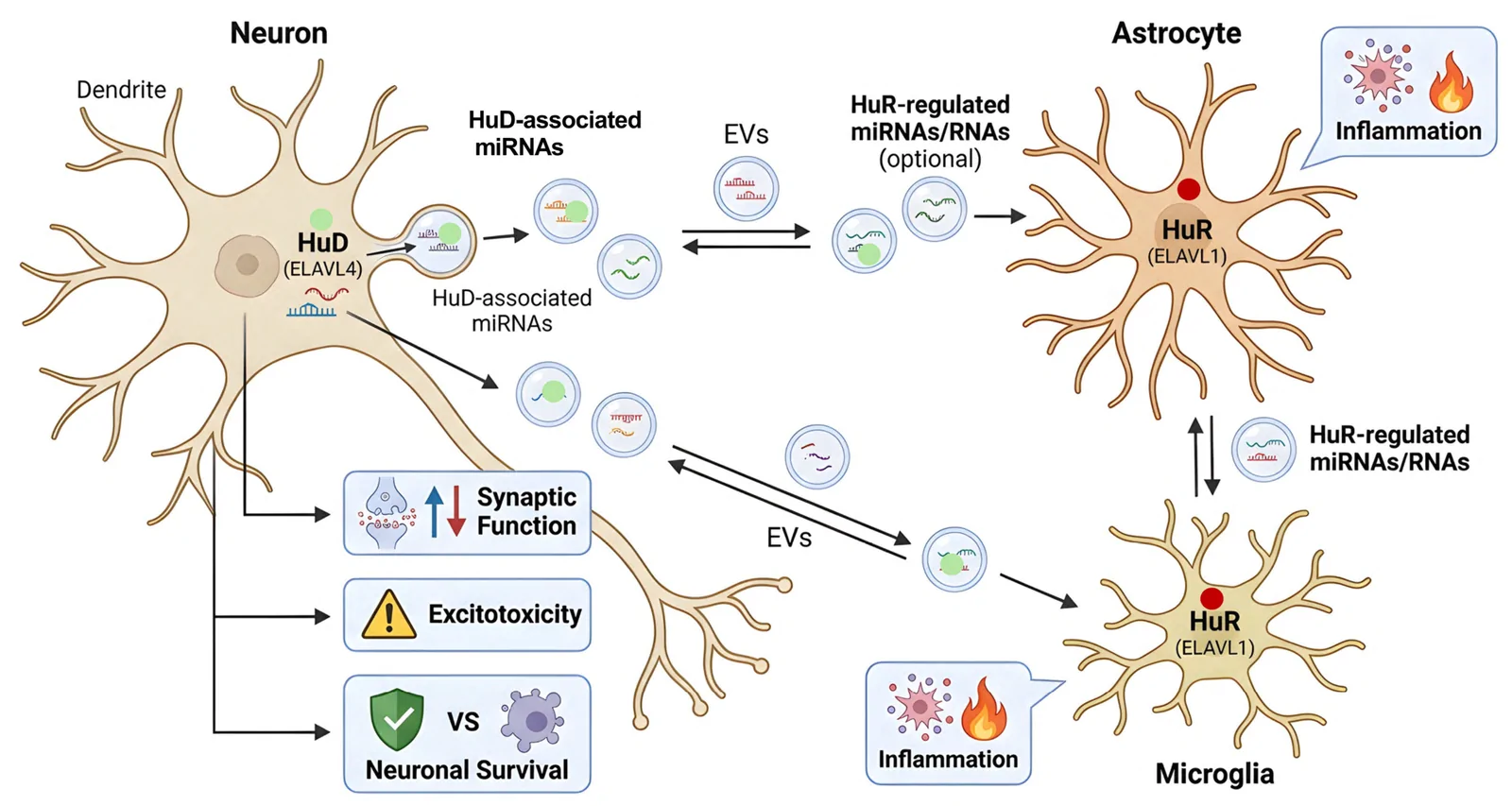
**ELAVL-Dependent Neuron–Glia miRNA Communication.** The illustration depicts ELAVL-dependent neuron–glia miRNA communication. It includes a neuron, an astrocyte, and a microglial cell participating in bidirectional extracellular vesicle exchange, highlighting HuD-associated miRNA selection in neurons and HuR-associated RNA regulation in glial cells.It also shows potential recipient-cell outcomes, including inflammation, altered synaptic function, excitotoxicity, and changes in neuronal survival. Arrows indicate the direction of EV-mediated communication and downstream cellular effects.

One hypothesis is that HuD-mediated export of repressive miRNAs from affected neurons enhances amyloidogenic gene expression and Aβ production, whereas transfer of these neuron-derived miRNAs to glia via EVs increases inflammation (Figure 2; Table 1 and Table 2). These apparently conflicting functions of HuD in AD require further investigation. Intercellular communication between neurons and glial cells, including astrocytes, microglia, and oligodendrocytes, is complex and essential for maintaining CNS homeostasis, synapse formation, and plasticity, which are likely affected in AD and PD. The transfer of miRNAs via extracellular vesicles (EVs), such as exosomes, constitutes a crucial regulatory pathway [83], and proteins from the ELAVL family (HuR and neuron-specific HuD) control the stability, transport, and activity of miRNAs, influencing both normal and disease processes [15,23].

Glial cells and neurons exchange miRNAs via EVs, affecting gene expression. Microglia release EVs containing miR-146a-5p that modulate synaptic proteins and neurotoxicity [84]. This process is likely modulated by HuR, the mTORC1 activator Rheb, and STX5 [80,85]. Neurons release miR-124a, which is taken up by astrocytes to regulate proteins such as GLT1, thereby reducing excitotoxicity [86]. In diseases such as AD, altered EV miRNA patterns and toxic protein release may promote disease progression, potentially involving HuD. HuR can stabilize transcripts or export miRNAs by competing at AU-rich elements, while another ELAVL protein, HuC, cooperates with miR-124 in neurogenesis [87].

Extracellular vesicles provide an established mechanism for intercellular RNA communication, and neuronal and glial cells can exchange regulatory miRNAs through EVs. However, whether the HuD-dependent miRNA-export mechanism identified in NGF-treated PC12 cells operates in vivo in the aging or neurodegenerative human brain remains unknown. In particular, there is currently insufficient evidence to conclude that HuD-dependent neuronal miRNA export is altered in AD neurons or that HuD-associated miRNAs are subsequently taken up by glial cells. We therefore consider the neuronal HuD–miRNA–EV pathway an emerging and testable mechanism rather than an established pathway of AD-associated neuron–glia communication.

**Figure 3:**
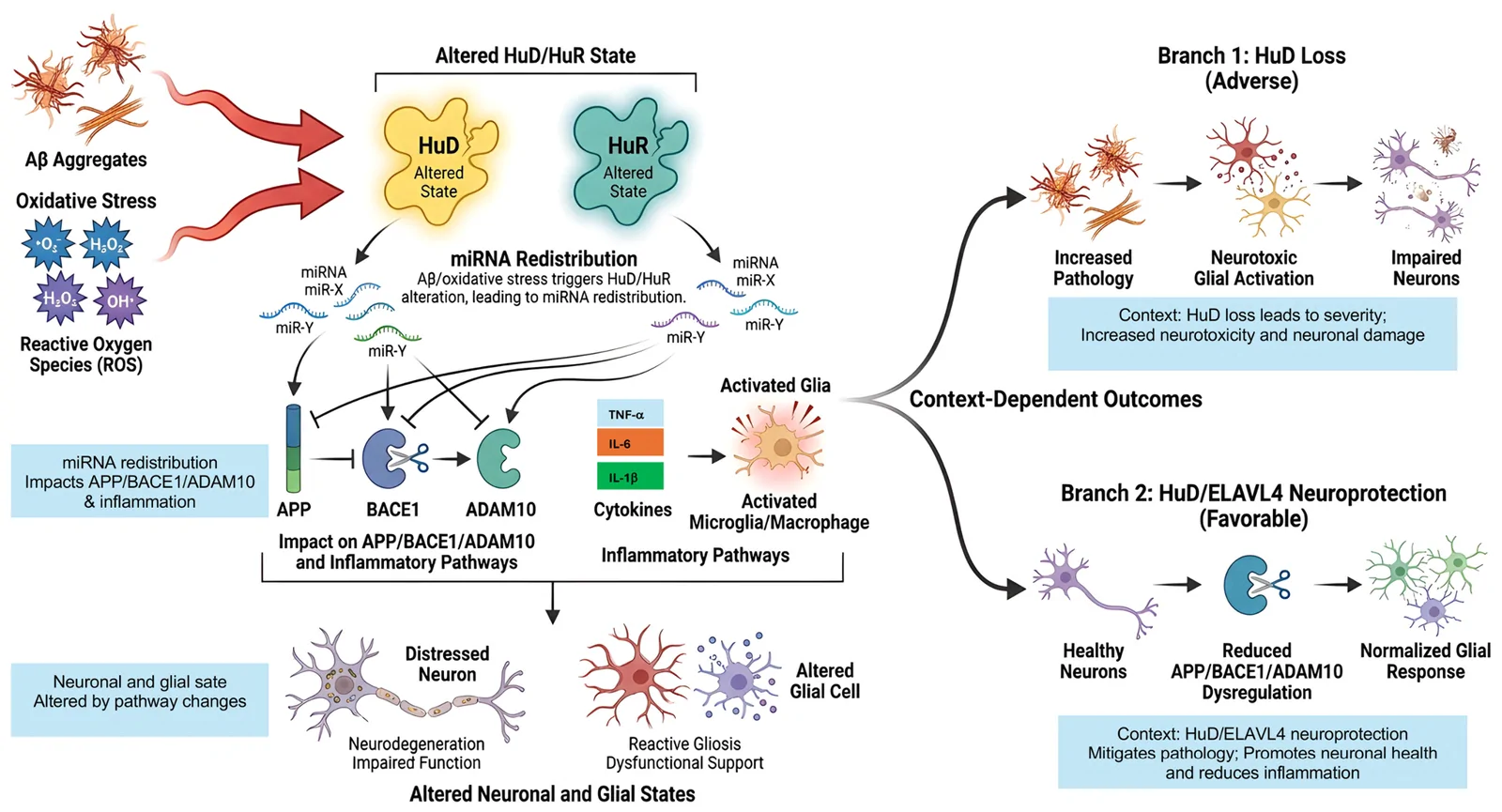
**ELAVL–miRNA axis in Alzheimer’s disease.** This illustration depicts the ELAVL–miRNA axis in Alzheimer’s disease and the complex interactions that may lead to altered cellular states. Stylized Aβ aggregates and reactive oxygen species represent initial stressors that contribute to disease progression. HuD and HuR icons represent altered protein states. miRNA trajectories illustrate how changes in HuD and HuR may influence their intracellular distribution. Molecular icons link these miRNA alterations to various pathways involved in Alzheimer’s disease, including inflammatory processes. The diagram also depicts neurons and glial cells in distressed and healthier states affected by these pathways. Two branches contrast the effects of HuD loss with HuD/ELAVL4-mediated neuroprotection, emphasizing the context-dependent nature of these mechanisms. Arrows indicate the proposed direction of regulatory effects and downstream outcomes; colors distinguish the molecular and cellular states as labeled in the figure.

Thus, ELAVL proteins clearly regulate RNA stability and translation, and model-specific studies support roles for HuD or HuR in the fate of selected miRNAs. Whether these activities govern vesicular miRNA packaging for glial communication in AD remains to be tested. HuD abundance may have context-dependent effects in AD models, while HuR and HuD could differentially influence inflammatory and neuronal-survival programs (Figure 3).

## 6. ELAVLs as Selective miRNA-Binding and Export Regulators

HuR stabilizes mRNAs and can bind and sequester particular miRNAs, including miR-21, thereby relieving repression of defined targets [88]. This substrate-specific activity should not be interpreted as broad stoichiometric “sponging” of cellular miRNAs. In NGF-differentiated PC12 cells, HuD associates with let-7a and miR-125b, reduces their intracellular abundance and activity, and increases their recovery in EVs [15]. These data establish a differentiation-associated export mechanism in that transformed neuronal model, but do not establish transfer to glial cells or operation of this mechanism in aging or AD. RNA immunoprecipitation supports HuD–miRNA association; direct binding, affinity, stoichiometry, and domain requirements remain to be tested with purified HuD and complementary cross-linking approaches [15,48]. HuR-dependent work indicates that selected miRNAs can be transferred through endosomal machinery involving RalA and Syntaxin 5 before extracellular export [16,23,85]. Other neuronal ELAVL proteins also intersect with miRNA pathways; for example, HuC and miR-124 cooperate in neuronal maturation [87]. Together, these studies justify testing whether ELAVL proteins regulate distinct miRNA sets across defined cell states, without assuming a universal export mechanism or recipient-cell effect (Figure 3; Table 2 and Table 3).

## 7. Brain Aging as a Transition in ELAVL-Dependent RNA Communication

Aging provides a biologically relevant context in which the ELAVL–miRNA network may become destabilized. Loss of HuD has been linked to increased expression of senescence-associated secretory factors, including CCL2, while recent work shows that HuD can protect neuronal cells against metabolic stress by sustaining PPARα expression and opposing miRNA-mediated repression. These findings indicate that HuD can couple RNA regulation to metabolic resilience and the inflammatory state. Conversely, altered HuR activity under stress can influence inflammatory transcripts, proteostasis, and RNA compartmentalization [89,90].

We propose, as a testable hypothesis rather than an established mechanism, that aging may alter the spatial and functional balance among ELAVL proteins. The rationale comes from separate observations that HuD abundance or function changes in neuronal differentiation, senescence-associated signaling, and disease models, whereas HuR responds to stress and regulates inflammatory transcripts or selected miRNA-export pathways in other cellular systems [15,16,18,79,90]. These component findings do not demonstrate coordinated ELAVL remodeling in the aging brain. Paired measurements of HuD/HuR abundance, localization, RNA interactomes, and intracellular and EV miRNA pools in age-matched neurons, astrocytes, and microglia are required to test whether cell-type-specific remodeling changes RNA communication.

**Figure 4:**
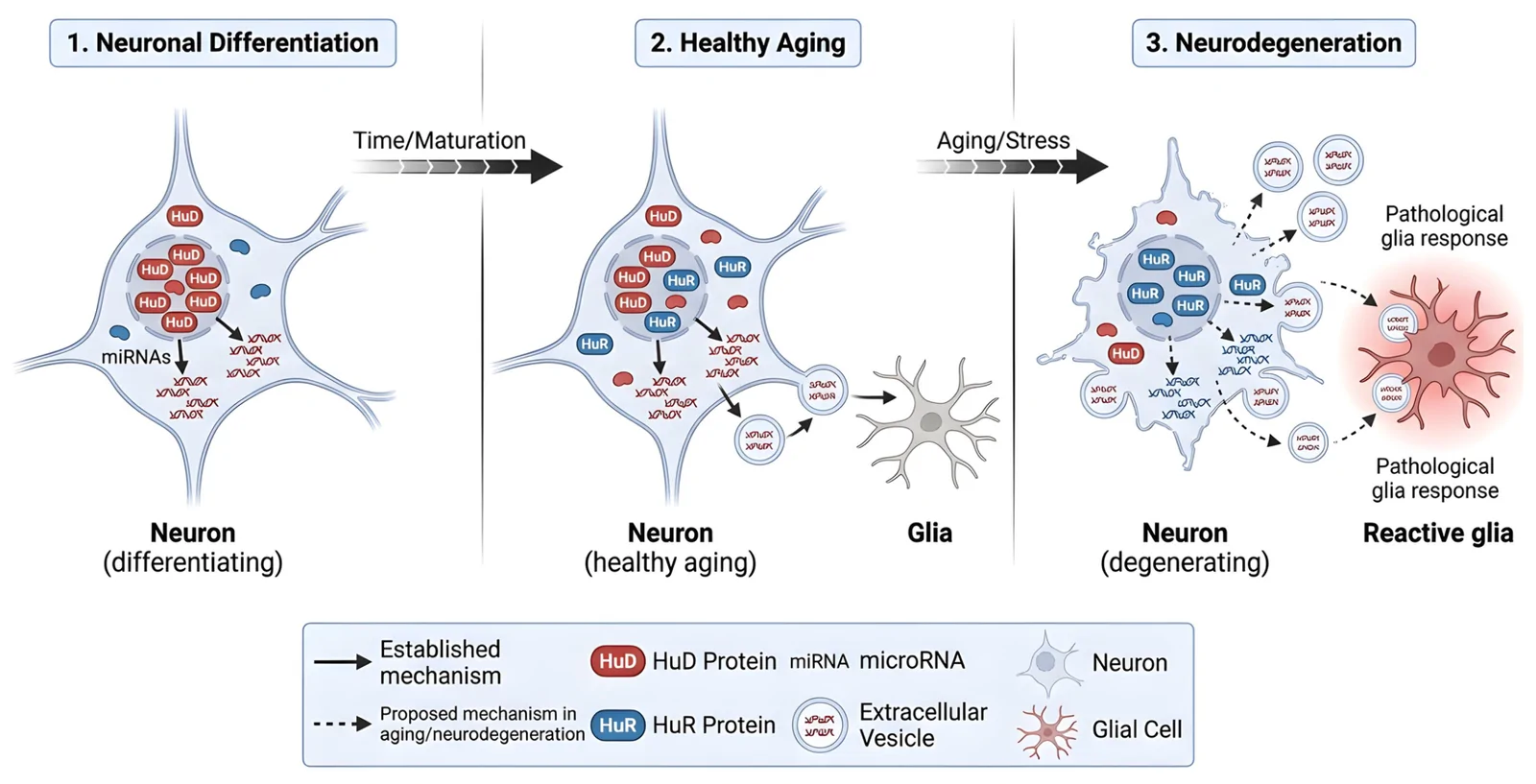
**Model-specific evidence and the proposed HuD–HuR miRNA-fate framework**. (**Left**): NGF-induced differentiation of PC12 cells is accompanied by increased HuD, decreased HuR, and enhanced export of let-7a and miR-125b [15]. This panel does not represent a “young neuronal state.” (**Middle**,**Right**): proposed, unproven changes in ELAVL localization or activity, miRNA pools, EV cargo, and neuron–glia signaling during aging and neurodegeneration. Solid arrows denote experimentally supported relationships in the stated model; dashed arrows denote hypotheses requiring validation.

This perspective also helps reconcile apparently contradictory observations concerning HuD in Alzheimer’s disease. HuD can support neuronal differentiation, synaptic function, and ADAM10 expression, yet HuD reduction in some settings can decrease amyloid pathology, while other models indicate that ELAVL4 activity can ameliorate AD-associated molecular abnormalities. These findings argue against a simple protective-versus-pathogenic classification. Instead, HuD function is likely determined by cellular context, RNA substrate selection, disease stage, and interaction with other ELAVL proteins (Figure 4, Table 4).

The ELAVL family (ELAVL1–4) supports brain health through molecular interactions, such as HuD’s role in glutamatergic signaling and synaptic plasticity. Abnormal expression in aging models can lead to neurodegeneration. In diseases such as ALS, early abnormalities such as nuclear depletion of ELAVL3 and TDP-43 pathology suggest that ELAVL dysregulation increases neuronal vulnerability [91]. HuD loss has been associated with increased production of senescence-associated secretory phenotype (SASP) factors, including CCL2; this supports a connection to senescence-associated inflammatory signaling but does not demonstrate accelerated cellular or brain aging [90]. HuR participates in stress responses involving the proteasome and autophagy; under stress, high levels of HuR can cause harmful protein aggregates if autophagy fails to clear them [89,92]. Aging also affects systemic communication, such as the heart–brain and muscle–brain axes, where RBPs help maintain balance. Disruptions in the heart–brain connection via the autonomic nervous system and blood vessels increase the risk of cardiovascular and neurodegenerative diseases. ELAVLs regulate miRNA export via EVs; their misexpression during aging, along with altered EV miRNA cargo, may impair communication with tissues such as the heart or gut [93]. This crosstalk may contribute to aging complications by misregulating target mRNAs through EV-mediated miRNA exchange between the CNS and peripheral tissues.

## 8. Alzheimer’s Disease as a Mechanistic Exemplar

Alzheimer’s disease provides a strong context in which to examine the ELAVL–miRNA model because amyloid pathology, RNA dysregulation, neuroinflammation, and aging converge. HuD regulates coding and non-coding RNAs involved in APP processing, while nELAVL alterations have been reported in the AD brain. Importantly, recent *in vivo* evidence shows that neuron-specific HuD ablation in 5xFAD mice reduces amyloid plaque burden and alleviates selected AD-associated behavioral abnormalities [12]. In contrast, studies in human iPSC-derived neurons have reported that ELAVL4/HuD expression can ameliorate AD-related molecular changes [82]. These apparently divergent observations should be treated as a central biological question rather than as a contradiction to be resolved by selecting one model.

One possibility is that HuD has stage- and cell-state-specific effects. HuD may promote APP/BACE1 expression in some neuronal contexts while supporting ADAM10, synaptic, or stress-resilience programs in others. At the same time, HuD-dependent miRNA sequestration and export could change the repression of multiple transcripts simultaneously. HuR may contribute a complementary mechanism in astrocytes and microglia by regulating inflammatory mRNAs and miRNA export. Thus, the relevant variable may be the ELAVL network state rather than the abundance of any single protein.

This framework generates experimentally testable predictions. First, HuD- and HuR-associated miRNA pools should differ between young and aged neurons and between neurons, astrocytes, and microglia. Second, amyloid exposure should alter the partitioning of these miRNAs between Ago-containing intracellular pools and EVs. Third, manipulating HuD/HuR levels should alter EV miRNA cargo and recipient-cell inflammatory or synaptic phenotypes. These studies would directly test whether the ELAVL–miRNA axis links aging-associated RNA dysregulation to AD progression.

### 8.1. The Knowledge Gap in Understanding the Role of ELAVL Proteins in AD

nELAV reduction has been associated with decreased ADAM10 expression, potentially impairing non-amyloidogenic APP processing because ADAM10 is the principal constitutive α-secretase in neurons [66,81]. nELAV proteins may also regulate AD-relevant genes through alternative splicing [14]. Based on published data showing the roles of HuR and HuD in controlling miRNA export and activity in non-neuronal and neuronal cells, we propose that HuR and nELAVs regulate miRNA export and function in brain cells. HuD, a member of the ELAVL family, has been associated with Aβ plaque formation in mice, yet other studies suggest the opposite. For example, decreased HuD levels have been observed with AD progression [66], and HuD has demonstrated protective effects in neurons derived from human iPSC lines [82]. Using a neuron-specific HuD knockout (HuDcKO) in the 5xFAD AD mouse model, Ji et al. discovered that Aβ plaque formation was reduced and AD-related hyperactivity was alleviated in 5xFAD/HuDcKO mice [12]. These findings emphasize the importance of investigating the roles of ELAVL proteins in AD and neuroinflammation at the molecular level, with a particular focus on miRNA–target mRNA regulation. Importantly, the role of individual ELAVs in glial and neuronal miRNA regulation and downstream pathways is unknown. HuD and other neuronal ELAVL proteins contain three RNA-recognition motifs with established RNA-binding functions; RRMIII contributes to poly(A)-tail recognition and should not be described as a miRNA-binding domain without direct domain-specific evidence. Although ELAVL proteins interact with selected miRNAs, the precise domains and molecular determinants governing these interactions and subsequent EV enrichment remain incompletely defined.

### 8.2. The Mechanism

Like HuR, neuronal ELAVL proteins contain three RNA-recognition motifs (RRMs), and these domains may contribute to interactions with selected RNAs; however, the domain-specific determinants of miRNA binding remain incompletely defined. Accordingly, HuR and neuronal ELAVL proteins may regulate distinct miRNA groups, but their context-dependent export and effects on inflammatory responses require direct testing (Figure 1 and Figure 3). Studies using primary neurons, glial cells, iPSC-derived brain organoids, and 5xFAD/ELAVLcKO mice should determine the miRNAs associated with HuR and nELAVLs. Manipulating ELAVL levels could identify which miRNAs each ELAVL regulates and assess their roles in EV-mediated miRNA export. In addition, experiments should examine how amyloid proteins in brain organoids and 5xFAD AD mice, which harbor familial human AD-linked mutations in the APP and PS1 genes, affect ELAVL-regulated miRNAs and identify key miRNAs associated with AD phenotypes. The possibility of reversing these effects by targeting specific ELAVL–miRNA interactions will need to be explored in the *in vivo* and *ex vivo* AD models.

### 8.3. ELAVL Proteins in HAND

HIV-associated neurocognitive disorders (HAND) affect 30–50% of people with HIV, even during treatment [94]. They involve neurodegeneration associated with chronic neuroinflammation, viral protein toxicity (such as gp120 and Tat), and synaptic damage [95]. Despite combination antiretroviral therapy (cART), HIV persists in the CNS, causing ongoing neuroinflammation and neuronal loss. HAND includes asymptomatic neurocognitive impairment (ANI), mild neurocognitive disorder (MND), and HIV-associated dementia (HAD), with symptoms such as memory impairment, attention deficits, executive dysfunction, and motor problems.

Viral proteins gp120 and Tat cause neuronal injury and sustained inflammation, leading to synapse loss. ELAVL proteins may protect neurons, but HIV may disrupts their functions, promoting neurodegeneration [96]. These proteins are essential for axonal and dendritic integrity, with dysfunction linked to synaptic impairment. HIV invades the CNS, causing damage via toxic proteins and microglial activation, worsening neurodegeneration. cART prevents severe dementia but does not fully prevent neuronal damage, indicating a need for new treatments [97]. Viral proteins affect the miRNA machinery and miRNA-regulatory subcellular structures, such as stress granules and P-bodies [98,99], which ELAVL proteins also influence [22,69]. HuR-binding sites on HIV-1 RNA suggest potential regulatory roles for HuR in HIV-1 gene expression [100,101]. Viral proteins also influence neuron–microglia interactions via EVs and associated miRNAs. Because ELAVL proteins can regulate selected miRNAs in model-specific settings, it will be important to test whether they affect HIV-1-associated miRNA networks, viral-protein responses, neuroinflammation, or neurodegeneration; direct evidence for this integrated mechanism is not yet available.

## 9. SMN and HuD: A Two-Faced Interaction

Motor neurons depend on the SMN protein and HuD to transport and stabilize specific mRNAs such as cpg15 and gap43 for axonal growth [7,8,102]. In healthy neurons, SMN and HuD interact via SMN’s Tudor domain to facilitate local translation at growth cones, thereby supporting axon branching and neuromuscular junction formation [102]. In spinal muscular atrophy (SMA), reduced SMN disrupts mRNA localization, leading to neurofilament accumulation and pathological granules at the neuromuscular junction, impairing transport and causing neuronal degeneration. Increasing HuD activity has shown promise in rescuing axonal defects and restoring movement in models of SMN deficiency. However, how SMN may affect HuR/HuD deficiency or dysfunction in the neurodegenerative contexts of AD and PD remains an interesting question [8].

SMN may directly affect or counteract deficiencies in HuR/HuD, especially in Alzheimer’s and Parkinson’s diseases, where these proteins are often sequestered or mislocalized, leading to the destabilization of key neuronal mRNAs. Since SMN, HuR, and HuD share overlapping roles, their interactions may involve competitive mRNA binding, regulation of miRNA activity, mitochondrial dynamics, and sequestration in pathological aggregates. SMN may buffer against HuR/HuD dysfunction by chaperoning shared target mRNAs, influencing miRNA-mediated silencing, and regulating mitochondrial health, potentially mitigating neuronal damage [103]. In disease contexts, SMN’s role in the assembly of stress granules may prevent abnormal aggregation of RBPs such as HuR and HuD, maintaining their functional fluidity [104]. Understanding these mechanisms offers mechanistic insight into neurodegeneration, with the potential to target SMN-related pathways for therapeutic benefit [105,106].

## 10. ELAVL-Controlled Extracellular miRNA Communication in Neurodegeneration

Extracellular vesicles provide a route by which neurons, astrocytes, and microglia exchange regulatory RNAs. EV-associated miRNAs can alter recipient-cell gene expression and thereby influence synaptic function, inflammatory signaling, and neuronal survival. Importantly, EV miRNA abundance is not necessarily a passive reflection of intracellular abundance; selective sorting mechanisms determine which RNAs enter EVs.

ELAVL proteins are candidates for participating in this selection. HuR can bind selected miRNAs and engage an export pathway involving RalA-dependent endosomal targeting and Syntaxin 5 in non-neuronal cellular models [16,85]. HuD promotes EV-associated export of let-7a and miR-125b during NGF-induced PC12 differentiation [15]. These model-specific results support testing a broader principle in which ELAVL proteins couple intracellular miRNA binding to extracellular RNA trafficking; they do not establish neuron-to-glia transfer in AD.

**Figure 5:**
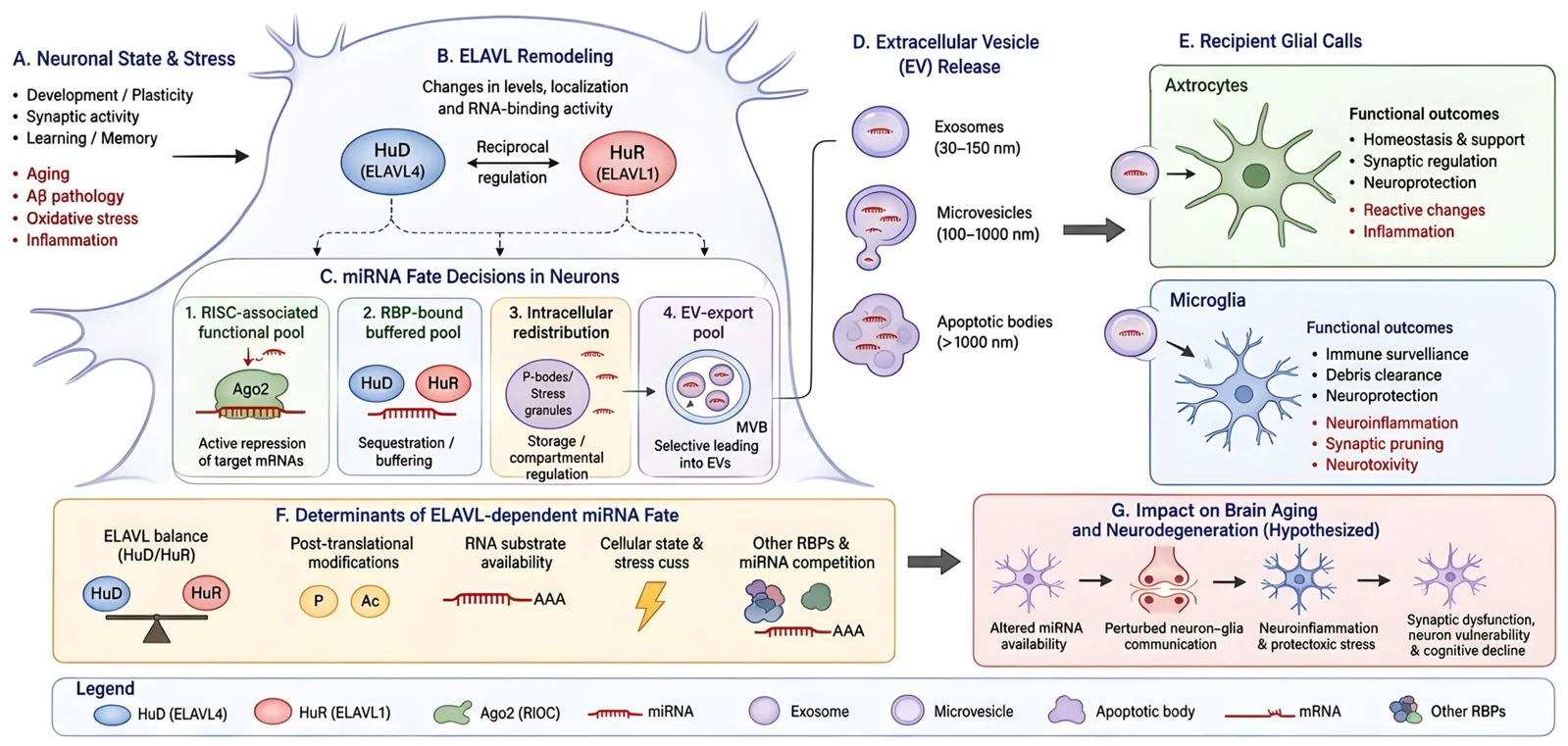
**ELAVL proteins (HuD and HuR) may remodel in response to neuronal state and stress.** (**A**,**B**) Candidate ELAVL changes. (**C**) Four possible miRNA fates: RISC-associated activity, RBP-mediated buffering, intracellular redistribution to granules or organelles, and EV export. (**D**,**E**) Potential cellular outcomes. (**F**) Candidate determinants of ELAVL-dependent miRNA fate. (**G**) Hypothesized consequences for neuroinflammation, synaptic dysfunction, and neurodegeneration. Red elements denote emerging or hypothetical steps. Arrows indicate the proposed flow from neuronal state and ELAVL remodeling to miRNA fate decisions, EV release, and recipient-cell outcomes; symbol colors are defined in the in-figure legend.

In neurodegenerative disease, this pathway could become maladaptive. Amyloid-associated stress, oxidative stress, and inflammatory signaling may alter ELAVL localization or RNA-binding activity, while disease-associated changes in EV production and cargo could modify communication between neurons and glia. A useful working model is that neuronal HuD and glial HuR regulate partially distinct miRNA pools, creating cell-type-specific EV signatures. However, the identity of these ELAVL-associated miRNAs, the molecular determinants of their sorting, and their functional effects in recipient cells remain incompletely defined (Figure 5).

## 11. Evidence Limitations, Unresolved Questions, and Therapeutic Opportunities

HuD has been implicated in both neuroprotective and potentially pathogenic processes, depending on model, disease stage, and RNA targets. In AD-related systems, HuD can stabilize APP- and BACE1-associated transcripts, whereas other findings link HuD to ADAM10 expression, neuronal resilience, or amelioration of selected disease-associated molecular changes [46,66,81,82]. Findings in experimental autoimmune encephalomyelitis likewise indicate context-dependent relationships among HuR inhibition, HuD abundance, neuroprotection, and pain rather than a simple HuD-deficiency model [79]. Compounds such as folic acid have been reported to alter HuD abundance, methylation, and BDNF expression in SH-SY5Y cells, but their relevance as HuD-directed therapies for neurodegeneration remains unestablished [103]. These observations argue for target- and context-selective intervention rather than global HuD activation or inhibition.

### 11.1. The HuD–HuR Switch Model and Its Evidentiary Boundary

The reciprocal behavior of HuD and HuR during neuronal differentiation exemplifies ELAVL-dependent miRNA regulation. In differentiating PC12 cells, HuR decreases as HuD rises, with HuD assuming a miRNA-export role previously linked to HuR. HuD binds let-7a and miR-125b, reducing their intracellular levels and activity while increasing their presence in EVs. This suggests ELAVL proteins can act as cell-state-dependent regulators of miRNA fate rather than static binders. A key question for brain aging is whether the HuD–HuR balance stays stable or shifts due to cellular stress, which involves oxidative stress, proteostasis defects, altered autophagy, inflammation, and extracellular communication. These factors can affect RNA-binding proteins and EV biogenesis, potentially altering miRNA availability and release. While direct evidence is lacking, mechanistic links are supported: HuR controls miRNA availability and export; HuD influences miRNAs in neuronal differentiation; HuD loss affects inflammatory signaling; and EV-associated miRNAs are linked to brain aging and neurodegeneration. Future research should aim to connect these findings experimentally (Figure 5, Table 5).

### 11.2. Limitations of the Available Evidence

The principal limitation is that the evidence is distributed across different experimental systems. The HuD-dependent export of let-7a and miR-125b was demonstrated during differentiation of PC12 cells, a rat pheochromocytoma-derived transformed line [15]. Although PC12 cells are useful for mechanistic studies, they do not reproduce the molecular diversity, maturity, or multicellular environment of primary human neurons. HuD modulation by folic acid was examined in SH-SY5Y neuroblastoma cells [103], and several HuR-dependent miRNA-trafficking mechanisms were established in non-neuronal cellular models [16,85]. Mouse and 5xFAD studies provide in vivo evidence for selected HuD-dependent phenotypes, but species differences and familial-AD transgenes limit extrapolation to sporadic human AD [12]. Finally, EV-associated RNA exchange between neural cell types is established in several settings, yet no study has linked, in one causal chain, ELAVL binding in a donor neuron, selective miRNA loading into EVs, uptake by a defined glial recipient, and a functional outcome in aging or AD. Conclusions throughout this review are therefore labeled as direct evidence, model-supported inference, or hypothesis. Validation will require primary human neurons and glia, iPSC-derived co-cultures and organoids, cell-type-resolved in vivo perturbations, and quantitative tests of EV uptake and recipient-cell target repression.

### 11.3. Unresolved Questions and Therapeutic Opportunities

Several questions now become central. Which miRNAs are directly bound by HuD, HuR, and other nELAVLs in aging neurons and glia? Does the HuD/HuR ratio change with age, amyloid burden, or inflammatory state? Which post-translational modifications determine ELAVL-dependent miRNA sorting? Does HuD remain associated with miRNAs throughout EV biogenesis, or are miRNAs transferred to dedicated sorting proteins before secretion? Which recipient-cell pathways are activated by ELAVL-dependent EV miRNAs? Finally, can selective modulation of ELAVL–miRNA interactions restore RNA homeostasis without disrupting the essential neuronal functions of HuD?

Therapeutically, the goal should not be global suppression or activation of HuD. A more precise strategy would target disease-associated ELAVL–RNA interactions, miRNA sorting, or EV communication while preserving physiological neuronal RNA regulation. Such approaches could include selectively disrupting pathogenic RBP–RNA interactions, modulating miRNA cargo sorting, or restoring disease-associated miRNA pools. These possibilities remain exploratory and require validation in primary human neurons and glia, induced pluripotent stem cell-derived systems, organoids, and *in vivo* models.

## 12. Conclusions

ELAVL proteins provide a plausible mechanistic bridge between intracellular RNA regulation and extracellular RNA communication. HuD and HuR share a related RNA-binding architecture but occupy distinct cellular and physiological contexts. Direct evidence shows HuD-dependent export of selected miRNAs during PC12 differentiation and HuR-dependent trafficking of selected miRNAs in other cellular models. Whether these mechanisms integrate into neuron–glia communication during human brain aging or neurodegeneration remains unresolved.

Our model therefore makes a bounded, testable proposal: age- or disease-associated changes in ELAVL abundance, localization, RNA binding, or interaction with miRNA machinery may redistribute selected miRNAs among intracellular regulatory pools and EVs. Demonstrating this mechanism will require cell-type-resolved studies that connect ELAVL binding to EV sorting and functional uptake in recipient cells. Framed in this way, ELAVL-dependent miRNA fate is an emerging research interface—not an established AD pathway—that may help explain context-dependent HuD effects and guide future studies of brain aging, neuroinflammation, and neurodegeneration.

## Figures and Tables

**Table 1:** ELAVL proteins and RNA regulatory functions relevant to brain aging and neurodegeneration.

ELAVL	Cellular/Model Context	RNA Function	Refs.
HuD/ ELAVL4	Neurons; neuronal tissues and neuronal models used to establish mRNA regulation	mRNA stability, translation, localization; neuronal RNA regulation	[1,2]
HuD/ ELAVL4	NGF-differentiated PC12 cells; evidence concerns differentiation-associated miRNA export, not in vivo neuron-to-glia transfer	miRNA binding and EV export	[15]
HuR/ ELAVL1	Broadly expressed; inflammatory and stress-responsive functions reported in multiple cell types, including macrophage models; expression also occurs in neurons, astrocytes, and microglia	mRNA stability/translation and stress responses	[16,17]
HuR/ ELAVL1	HuR-dependent miRNA binding and endosomal export established in non-neuronal cell systems; Aβ-linked miRNA compartmentalization examined in cellular models, without proof of neuron-to-glia transfer in AD	miRNA buffering and EV export	[16,18]
HuB/HuC	Predominantly neurons; functions supported by neuronal tissue and neuronal-model studies	RNA processing/regulation	[2]

**Table 2:** mRNAs and RNAs regulated by HuD in neurodegenerative-disease models.

Target RNA	Disease	Mode of Regulation by HuD	Role in Pathogenesis
APP (Amyloid Precursor Protein)	AD	Binding to 3′-UTR, stabilizing mRNA	Increases amyloid-β (Aβ) peptide production [6,46,66]
BACE1 (β-site APP-cleaving enzyme 1)	AD	Binding to 3′-UTR, stabilizing mRNA	Increases β-secretase, leading to higher Aβ levels [46,66]
BACE1AS (BACE1 antisense lncRNA)	AD	Binding and stabilization of lncRNA	Promotes BACE1 expression and amyloidogenesis [46,66]
Tau (MAPT)	AD	Binding and stabilizing mRNA	Potential role in tau protein accumulation [5]
Neuroserpin	AD	Stabilization of mRNA	Inhibits tPA; increased levels may reduce Aβ degradation [44]
SOD1 (Superoxide dismutase 1)	PD/ALS	Stabilization of mRNA	Implicated in oxidative stress pathways [68]
cirHomer1a (Circular RNA)	General/AD	Binding and regulation of expression	Affects synaptic plasticity [54]
GAP-43 (Growth-Associated Protein)	General	Stabilization of mRNA	Neuronal remodeling and plasticity [3,59,64]

Abbreviations: AD, Alzheimer’s disease; ALS, amyotrophic lateral sclerosis; APP, amyloid precursor protein; Aβ, amyloid-β; BACE1, β-site APP-cleaving enzyme 1; GAP-43, growth-associated protein 43; lncRNA, long non-coding RNA; PD, Parkinson’s disease; SOD1, superoxide dismutase 1.

**Table 3:** ELAVL-dependent miRNA fate: evidence and proposed model.

State	Mechanism and Implicated miRNA(s)	Outcome	Evidence Status	Refs.
Neuronal differentiation	HuD increases as HuR decreases; HuD binds let-7a and miR-125b and promotes their EV export	Reduced intracellular let-7a/miR-125b activity supports differentiation	Direct evidence	[15]
Cellular stress	HuR can bind selected miRNAs and regulate intracellular/export fate; the relevant miRNA set in aging neurons is not established	Adaptive reduction/redistribution of selected miRNAs	Direct evidence in non-neuronal stress models	[16,88]
Aβ-associated stress	Aβ enhances Ago2/miR-146a P-body targeting; HuR can disrupt miRNA compartmentalization; a broader ELAVL-bound set is not defined	Changes miRNA compartmentalization	Direct mechanistic evidence	[18]
Brain aging	Potential remodeling of HuD/HuR abundance, localization, and RNA binding; implicated miRNAs are not established	Predicted redistribution of miRNAs between intracellular pools and EVs	Proposed hypothesis	[1,89]
Neurodegeneration	ELAVL remodeling may alter miRNA availability and EV cargo; implicated miRNAs are not established	Potential changes in neuron–glia signaling	Proposed hypothesis	[16,18,90]

**Table 4:** ELAVL–miRNA mechanisms at the neuron–glia interface.

Cell Type	ELAVL Focus	Potential miRNA/EV Mechanism	Disease Relevance	Refs.
Neuron	HuD/ELAVL4	Selective miRNA binding and EV export	May alter extracellular RNA signals during neuronal stress/aging	[15]
Astrocyte	HuR/ELAVL1	Stress-responsive RNA regulation; possible miRNA trafficking	Potential inflammatory/metabolic communication	[16]
Microglia	HuR/ELAVL1	miRNA/endosomal export mechanisms	Potential regulation of inflammatory activation	[15]
Neuron ↔ glia	HuD/HuR network	EV-mediated miRNA exchange	Candidate mechanism linking aging to neuroinflammation	[16,18,90]

**Table 5:** ELAVL/HuD/HuR evidence in aging and neurodegeneration.

Condition	Key Finding	Interpretation	Refs.
Brain aging/senescence	HuD loss increases senescence-associated secretory factors including CCL2; HuD binds Ccl2 mRNA	Supports a link between HuD-dependent RNA regulation and senescence-associated inflammatory signaling	[90]
Metabolic stress/senescence	HuD loss sensitizes neuroblastoma cells to palmitate-driven premature senescence through PPARα/FAO impairment	Supports a broader aging/stress connection; not yet direct evidence of brain aging	[90]
Alzheimer’s disease	HuD reduction alleviates AD pathology in 5xFAD mice	Demonstrates context-dependent disease effects of HuD	[12]
Alzheimer’s disease	HuR counteracts Aβ-induced Ago2/miRNA P-body targeting	Links HuR, miRNA compartmentalization and Aβ stress	[18]
Neuronal differentiation	HuD assumes a HuR-like miRNA export function	Provides mechanistic precedent for ELAVL switching of miRNA fate	[15]

## Data Availability

No new data were created or analyzed in this study. Data sharing is not applicable to this article.

## Abbreviations

Aβ, amyloid-β; AD, Alzheimer’s disease; Ago2, Argonaute 2; ALS, amyotrophic lateral sclerosis; APP, amyloid precursor protein; BACE1, β-site APP-cleaving enzyme 1; BDNF, brain-derived neurotrophic factor; cART, combination antiretroviral therapy; CCL2, C-C motif chemokine ligand 2; CNS, central nervous system; EAE, experimental autoimmune encephalomyelitis; ELAVL, embryonic lethal abnormal vision-like; EV, extracellular vesicle; HAND, HIV-associated neurocognitive disorder; HuC, ELAVL3; HuD, ELAVL4; HuR, ELAVL1; iPSC, induced pluripotent stem cell; miRNA, microRNA; miRISC, miRNA-induced silencing complex; MVB, multivesicular body; nELAVL, neuronal ELAVL protein; P-body, processing body; RBP, RNA-binding protein; RISC, RNA-induced silencing complex; RRM, RNA-recognition motif; SASP, senescence-associated secretory phenotype; SMA, spinal muscular atrophy; SMN, survival motor neuron; STX5, Syntaxin 5.
